# The effect of alternative permutation testing strategies on the performance of multifactor dimensionality reduction

**DOI:** 10.1186/1756-0500-1-139

**Published:** 2008-12-30

**Authors:** Alison A Motsinger-Reif

**Affiliations:** 1 Department of Statistics, Bioinformatics Research Center, North Carolina State University, Raleigh, North Carolina, USA

## Abstract

**Background:**

Multifactor Dimensionality Reduction (MDR) is a novel method developed to detect gene-gene interactions in case-control association analysis by exhaustively searching multi-locus combinations. While the end-goal of analysis is hypothesis generation, significance testing is employed to indicate statistical interest in a resulting model. Because the underlying distribution for the null hypothesis of no association is unknown, non-parametric permutation testing is used. Lately, there has been more emphasis on selecting all statistically significant models at the end of MDR analysis in order to avoid missing a true signal. This approach opens up questions about the permutation testing procedure. Traditionally omnibus permutation testing is used, where one permutation distribution is generated for all models. An alternative is *n*-locus permutation testing, where a separate distribution is created for each *n*-level of interaction tested.

**Findings:**

In this study, we show that the false positive rate for the MDR method is at or below a selected alpha level, and demonstrate the conservative nature of omnibus testing. We compare the power and false positive rates of both permutation approaches and find omnibus permutation testing optimal for preserving power while protecting against false positives.

**Conclusion:**

Omnibus permutation testing should be used with the MDR method.

## Background

One of the main goals of genetic epidemiology is the identification and characterization of polymorphisms that present an increased risk of disease. It is increasingly assumed that complex diseases are the result of a myriad of genetic and environmental risk factors [1,2]. This complex etiology limits the utility of traditional, parametric statistical approaches in genetic association studies [3,4]. The ubiquitous nature of gene-gene and gene-environment interactions [1,5,6] has inspired the development the novel statistical approaches designed to detect epistasis [7-9].

Multifactor Dimensionality Reduction (MDR) is one such method [10]. MDR was designed to detect interactions in categorical independent variables and a dichotomous dependent variable (*i.e*. case/control status or drug treatment response/non-response). MDR performs an exhaustive search of all possible single-locus through *n*-locus interactions (as computationally feasible) to evaluate all possible high/low risk models of disease. MDR selects a single model as optimal for each *n*-locus interaction as a result of these evaluations. Permutation testing (PT) is used to determine the significance of these models. MDR is nonparametric and model-free, so no hypotheses concerning the value of any statistical parameter nor any genetic inheritance model are made [10]. MDR has successfully identified interactive effects in simulated data as well as real data applications in diseases such as hypertension [3,11,12], cancer [10,13,14], and atrial fibrillation [15,16].

The end-goal of an MDR analysis is ultimately hypothesis generation (or refinement within candidate gene strategies) [17]. Hypothesis testing is used within the MDR analysis framework to determine whether resulting models are significantly different than expected by chance. Significance of a model is intended to indicate an interesting model that should be followed up in replication cohorts or functional studies. In recent work, there has been more emphasis on selecting all statistically significant models [17] in order to avoid missing a true signal (false negatives) in exchange for risking the selection of a few false positives. This generation of multiple hypotheses opens up questions about the PT procedure used to ascribe significance to this end set of models.

PT is a commonly used non-parametric statistical procedure that involves re-sampling the data without replacement to actually construct the distribution of the test statistic under the null hypothesis rather than make specific distributional assumptions. If the value of the test statistic based on the original samples is extreme relative to this distribution (i.e. if it falls far into the tail of the distribution), then the null hypothesis is rejected [18]. Validity of PT relies only on the property of exchangeability under the null hypothesis – that the joint distribution of the data samples must remain invariant to permutations of the data subscripts. Thus, permutation tests maintain a wide applicability under a much broader range of data and research conditions than most parametric tests [19]. In addition, PT requires minimal assumptions about the data being examined, yet often has power equal to, or even greater than, parametric counterparts that require stronger, and sometimes untenable data assumptions [20]. Unlike many parametric and other nonparametric tests, the results of permutation tests (the p-values) are unbiased [18]. The chief drawback of this method is that it is computationally expensive, but the easy availability of fast computing has made this a practical approach even for large datasets.

MDR implements PT to statistically test to the best model(s) [21]. Typically, omnibus PT is used, where a single null distribution is generated from the best model of each of at least one thousand randomized datasets. With a focus on selecting all potentially interesting models from the final MDR set, this omnibus method may be too conservative. *n*-locus PT is an alternative, where a separate null distribution is created for each *n*-level of interaction. So if single-locus through five-way interactions were evaluated in an original MDR analysis, a separate distribution would be created for the single-locus model, for the two-locus model, etc (for a total of five null distributions).

Currently, we compare the significance cut-offs, power, and false positive rates of omnibus PT and *n*-locus PT implemented in MDR for a wide range of disease models. We also examine the overall false positive rate of the MDR method using both types of PT. As the MDR method gains acceptance and is increasingly used in the genetics community, it is important that users understand how to properly apply PT.

### Multifactor Dimensionality Reduction (MDR)

**Figure 1:**
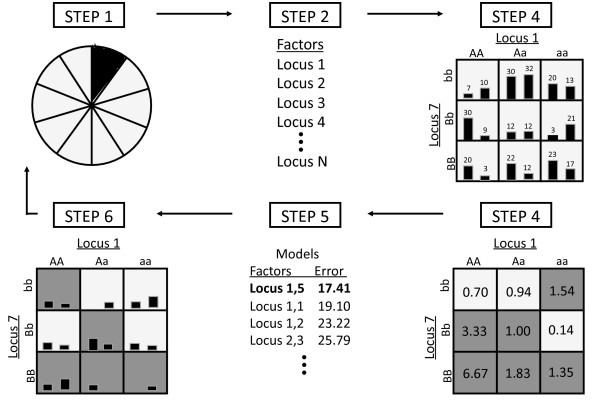
**An overview of the MDR method**. Steps correspond to those described in the supplemental information.

Figure 1 (adapted from [10]) outlines the MDR procedure. Details of the algorithm and of the alternative PT strategies implemented in the current study can be found in Additional file 1.

### Data Simulations and Analysis

Simulated datasets that exhibit gene-gene interactions were generated for the purpose of evaluating the power and false positives of MDR using either omnibus or *n*-locus PT. Multiple disease models, as well as null data with no disease model, were generated with varying allele frequencies, heritability, and number of interacting functional polymorphisms. Details of the simulations and analysis are found in Additional file 1.

## Results

Null data was used to check the false positive rates of both permutation-testing strategies in the absence of any signal from the data. Best models were chosen for each dataset based on low prediction error and high cross-validation consistency and compared to the appropriate permutation distribution. Table 1 shows the false positive rate for each permutation distribution, with alpha = 0.05, where the false positive rate is estimated as the number of models that were declared significant by PT out of the 100 datasets analyzed. These results demonstrate that the false positive rate is nominal for each permutation distribution – the error rate is at or below the selected alpha level.

**Table 1:** False positive rate for null data

Permutation Distribution False Positive Rate (%)					
l Locus	2 Locus	3 Locus	4 Locus	5 Locus	Omnibus
0	2	3	4	2	0

Both omnibus and *n*-locus permutation distributions were created for each model, and the highest prediction error that would be ascribed statistical significance at the alpha = 0.05 level was recorded (cut-offs for significance). Table 2 lists these cut-offs for omnibus testing and each possible *n*-locus distribution. For each model, the most conservative cut-off is highlighted with bold font. These results demonstrate that omnibus PT consistently provides the most conservative PT cut-offs, as its cut-off prediction errors are the lowest. There is also a general trend within the *n*-level PT distributions that as the level of *n *increases, so does the corresponding cut-off value. This demonstrates that the *n*-locus PT becomes more liberal as the level of dimensionality increases.

**Table 2:** Permutation testing significance cut-offs.

Epistasis Model			Permutation Distribution Cut-Off Prediction Error (%)					
Number of Functional Loci	Minor Allele Frequency	Heritability	1 Locus	2 Locus	3 Locus	4 Locus	5 Locus	Omnibus

2	0.2	3%	43.5	43.71	44.48	**43.42**	44.19	43.5
2	0.2	2%	43	42.5	43.83	43.84	43.82	**41.25**
2	0.2	1.5%	43.75	42.85	43.73	43.64	43.45	**42.5**
2	0.2	1%	43.5	42.75	44.12	45.05	44.24	**41.84**
2	0.2	0.5%	43.5	43.25	43.19	42.68	43.68	**40.75**
2	0.4	3%	42.75	43.75	44.12	43.15	44.22	**41.02**
2	0.4	2%	44.5	44.75	45	44.55	43.77	**42.25**
2	0.4	1.5%	44.25	43.5	42.46	42.34	43.62	**40.25**
2	0.4	1%	43	43.75	42.5	44.53	44.28	**40.25**
2	0.4	0.5%	43.5	43.25	43.31	44.2	43.2	**41.5**
3	0.2	3%	43.25	43.36	43.94	43.92	43.96	**42.75**
3	0.2	2%	43.25	42.5	44.09	44.14	45.71	**41.5**
3	0.2	1.5%	42.75	43.75	46.08	45.34	44.2	**42.25**
3	0.2	1%	42.75	43.75	43.85	43.47	44.36	**42.5**
3	0.2	0.5%	43.5	43.25	42.79	42.9	43.28	**42.5**
3	0.4	3%	43.25	43	45.5	45.74	45.15	**42.5**
3	0.4	2%	42.5	45	43.75	43.14	44.2	**42.25**
3	0.4	1.5%	42.75	45.25	44.22	45.56	43.74	**42.5**
3	0.4	1%	44.5	43	44.25	44.79	42.89	**41.5**
3	0.4	0.5%	43.25	44.5	43.86	44.92	44.5	**42.09**
4	0.2	3%	44.5	**41.5**	43.86	43.39	44.37	**41.5**
4	0.2	2%	43	42.6	43.29	45.76	45.13	**42.5**
4	0.2	1.5%	42.5	42.85	43.4	42.54	43.02	**40.59**
4	0.2	1%	43.75	43.1	43.16	44.07	44.15	**41.22**
4	0.2	0.5%	44	44	43.56	46.65	43.85	**42.97**
4	0.4	3%	44.5	43.25	44.49	44.03	45.2	**41.07**
4	0.4	2%	42.25	43.5	43.84	43.66	44.18	**40.75**
4	0.4	1.5%	42.25	43.5	45.25	44.08	42.64	**41.49**
4	0.4	1%	**41.5**	45	43.25	44.05	44.2	**41.5**
4	0.4	0.5%	41.75	44	44.85	44.4	43.41	**41**
5	0.2	3%	44.25	42.25	44.85	43.75	45.43	**41**
5	0.2	2%	42.75	43.5	44.63	45.32	46.04	**41.05**
5	0.2	1.5%	43.25	41.5	43.57	44.88	44.35	**40.75**
5	0.2	1%	43.75	44.62	45.1	43.54	45.06	**41.71**
5	0.2	0.5%	44	43.62	44.25	44.05	44.57	**40.08**
5	0.4	3%	43.5	42.5	43.75	43.99	43.11	**41.25**
5	0.4	2%	43.25	44	44.5	44.51	44.23	**40.78**
5	0.4	1.5%	45	43.5	44.97	44.96	43.83	**42.5**
5	0.4	1%	42.25	42.5	44.75	45.32	43.43	**40.75**
5	0.4	0.5%	44	43.25	43.5	45.14	45.01	**39.75**

While the anti-conservative nature of *n*-locus PT could potentially increase power, it is undesirable if that results in an increased false positive rate. To evaluate this, we investigated the false positive rate of each *n*-locus permutation distribution for each model. MDR analysis was performed on each dataset for all single-locus through five-locus combinations, and a best model was chosen for each level of interaction. For each dataset, the best model for each level of interaction was compared to the appropriate *n*-locus permutation distribution to estimate the false positive rate where a false positive result was any model that is not correct (may contain only incorrect loci or correct loci with additional false positive loci) and was found statistically significant according to the appropriate permutation distribution. Summarized in Table 3, using this definition of power, the false positive rate is extremely high for any *n*-level interaction above the true genetic model. For example, for the two-locus interaction model with 0.2 minor allele frequency and 3% heritability, all three, four, and five locus models were statistically significant.

**Table 3:** False positive rates for n-locus permutation distributions.

Epistasis Model			N-Locus Permutation Distribution False Positive Rate (%)				
Number of Functional Loci	Minor Allele Frequency	Heritability	1 Locus	2 Locus	3 Locus	4 Locus	5 Locus

2	0.2	3%	4	0	100	100	100
2	0.2	2%	2	0	84	76	62
2	0.2	1.5%	3	0	89	78	57
2	0.2	1%	3	1	50	55	33
2	0.2	0.5%	3	1	17	5	6
2	0.4	3%	4	0	95	89	76
2	0.4	2%	7	0	96	93	69
2	0.4	1.5%	7	0	82	57	45
2	0.4	1%	4	1	31	24	19
2	0.4	0.5%	6	3	6	13	8
3	0.2	3%	3	5	0	100	100
3	0.2	2%	2	36	0	100	94
3	0.2	1.5%	2	65	33	67	48
3	0.2	1%	4	40	22	22	22
3	0.2	0.5%	2	1	1	3	3
3	0.4	3%	6	10	0	100	93
3	0.4	2%	5	46	2	57	46
3	0.4	1.5%	3	43	4	61	26
3	0.4	1%	11	12	13	17	4
3	0.4	0.5%	5	10	5	14	6
4	0.2	3%	9	46	85	0	95
4	0.2	2%	4	31	64	4	90
4	0.2	1.5%	1	18	26	11	20
4	0.2	1%	6	5	6	8	18
4	0.2	0.5%	6	9	8	23	11
4	0.4	3%	6	16	63	7	77
4	0.4	2%	0	7	24	3	23
4	0.4	1.5%	4	5	15	3	5
4	0.4	1%	1	14	7	4	12
4	0.4	0.5%	2	11	9	9	3
5	0.2	3%	4	21	51	64	21
5	0.2	2%	1	10	27	64	23
5	0.2	1.5%	1	1	6	6	9
5	0.2	1%	7	14	20	18	13
5	0.2	0.5%	3	2	3	3	5
5	0.4	3%	7	5	11	16	6
5	0.4	2%	1	6	6	12	7
5	0.4	1.5%	7	3	7	8	5
5	0.4	1%	0	2	10	10	6
5	0.4	0.5%	10	6	3	4	6

To better understand this trend, we estimated power for each model as the number of times all functional loci (with or without additional/false positive loci) were identified within the best model for any *n*-level interaction and was called significant according to the corresponding permutation distribution out of the 100 simulated datasets per model. Table 4 summarizes these results. These results suggest that the false positive rates shown in Table 3 may be driven by the inclusion of functional loci in higher-level interactions. This trend was seen for all models, but is especially apparent in higher heritability models. This suggests, especially in the case of a relatively strong signal from the data, that even containing the correct loci within the model is enough to drive it to statistical significance according to a more liberal PT procedure. This is a highly likely explanation, especially considering the nominal false positive rates demonstrated for null data (Table 1).

**Table 4:** Power (with or without additional loci) for n-locus permutation distributions.

Epistasis Model			N-Locus Permutation Distribution Power (%)				
Number of Functional Loci	Minor Allele Frequency	Heritability	1 Locus	2 Locus	3 Locus	4 Locus	5 Locus

2	0.2	3%	0	100	100	100	100
2	0.2	2%	0	100	95	92	89
2	0.2	1.5%	0	91	97	92	89
2	0.2	1%	0	55	80	85	74
2	0.2	0.5%	0	18	45	35	41
2	0.4	3%	0	100	97	93	86
2	0.4	2%	0	100	100	100	92
2	0.4	1.5%	0	100	100	92	80
2	0.4	1%	0	67	73	64	51
2	0.4	0.5%	0	12	40	30	28
3	0.2	3%	0	0	100	100	100
3	0.2	2%	0	0	100	100	100
3	0.2	1.5%	0	0	46	58	55
3	0.2	1%	0	0	2	24	39
3	0.2	0.5%	0	0	1	16	20
3	0.4	3%	0	0	100	100	100
3	0.4	2%	0	0	70	86	67
3	0.4	1.5%	0	0	65	65	39
3	0.4	1%	0	0	10	32	15
3	0.4	0.5%	0	0	2	12	8
4	0.2	3%	0	0	0	100	10
4	0.2	2%	0	0	0	91	10
4	0.2	1.5%	0	0	0	15	4
4	0.2	1%	0	0	0	9	4
4	0.2	0.5%	0	0	0	1	4
4	0.4	3%	0	0	0	82	11
4	0.4	2%	0	0	0	29	6
4	0.4	1.5%	0	0	0	12	4
4	0.4	1%	0	0	0	3	1
4	0.4	0.5%	0	0	0	1	0
5	0.2	3%	0	0	0	0	67
5	0.2	2%	0	0	0	0	49
5	0.2	1.5%	0	0	0	0	0
5	0.2	1%	0	0	0	0	22
5	0.2	0.5%	0	0	0	0	0
5	0.4	3%	0	0	0	0	18
5	0.4	2%	0	0	0	0	2
5	0.4	1.5%	0	0	0	0	1
5	0.4	1%	0	0	0	0	1
5	0.4	0.5%	0	0	0	0	1

Table 4 shows that the power of MDR is relatively high in lower order models, especially at the *n*-locus level of analysis. Interpretation of an MDR analysis is complicated, however, when using *n*-locus PT by the high level of false positives. Even though the functional loci are included in the significant models, choosing the correct order of interaction is difficult when *n*-locus PT is used for each level of interaction.

Understanding that omnibus PT is the more conservative option, we investigated its impact on both the overall power and false positive rates of the MDR method. Table 5 summarizes these results for each disease model. First, we wanted to compare the power of MDR to detect the correct model as the final model (through minimization of prediction error and maximization of cross validation consistency) without considering statistical significance. Power was estimated as the number of times the functional/disease associate loci were chosen as the best model across the 100 replicates, with no false positive loci included in the model. By defining power in this context (with no PT), this estimate represents the least conservative estimate. These results are equivalent to the least conservative cut-off possible with *n*-locus PT. By defining power in this way, by using any significance testing the power cannot possibly be higher – all results that count towards "power" under this definition can only be changed to non-significant by using any significance testing. Results are summarized in the column of Table 5 labeled "Power Without Permutation Testing". This is then compared to the power of MDR to not only find the correct model, but to also ascribe statistical significance to that model through omnibus PT. Results of this evaluation are presented in the column of Table 5 labeled "Power With Permutation Testing". Comparing the power with and without permutation demonstrates the results are similar. Omnibus PT does not severely limit the power of the method.

**Table 5:** Power of MDR with and without omnibus permutation testing and false positive rate with permutation testing.

Epistasis Model					
			
Number of Functional Loci	Minor Allele Frequency	Heritability	Power (%) Without Permutation Testing	Power (%) With Permutation Testing	False Positive Rate (%) With Permutation Testing
2	0.2	3%			
2	0.2	2%	90	88	0
2	0.2	1.5%	94	91	1
2	0.2	1%	70	56	6
2	0.2	0.5%	18	14	4
2	0.4	3%	93	93	2
2	0.4	2%	93	92	5
2	0.4	1.5%	95	91	1
2	0.4	1%	85	71	6
2	0.4	0.5%	25	11	0
3	0.2	3%	69	69	2
3	0.2	2%	85	85	2
3	0.2	1.5%	20	18	5
3	0.2	1%	6	2	6
3	0.2	0.5%	6	2	4
3	0.4	3%	89	89	1
3	0.4	2%	55	54	3
3	0.4	1.5%	40	34	2
3	0.4	1%	6	4	2
3	0.4	0.5%	3	0	1
4	0.2	3%	68	68	4
4	0.2	2%	59	57	8
4	0.2	1.5%	11	10	4
4	0.2	1%	10	9	2
4	0.2	0.5%	1	0	1
4	0.4	3%	60	52	1
4	0.4	2%	36	25	5
4	0.4	1.5%	4	4	7
4	0.4	1%	3	2	6
4	0.4	0.5%	0	0	3
5	0.2	3%	35	35	3
5	0.2	2%	3	3	3
5	0.2	1.5%	1	0	2
5	0.2	1%	20	11	4
5	0.2	0.5%	0	0	1
5	0.4	3%	15	10	0
5	0.4	2%	3	2	6
5	0.4	1.5%	0	0	9
5	0.4	1%	0	0	0
5	0.4	0.5%	0	0	5

Finally, we evaluated the impact of omnibus PT on the false positive rate. The false positive rate was estimated for each model as the number of incorrect final models that were statistically significant using omnibus PT. This calculation included significance testing at each level of interaction – not just a single test for one overall best model. As Table 5 shows, the false positive rates are near the expected 5% level.

From the results presented in Table 5, we conclude that omnibus PT controls for false positives while preserving power.

## Conclusion

In this study we confirmed that the overall false positive rate of MDR is as expected according to the selected alpha level. Additionally, we demonstrated the conservative nature of omnibus testing in comparison to an *n*-locus strategy.

We also demonstrated that omnibus PT is preferred to *n*-locus since it controls false positives without limiting power. While MDR has high power using either permutation-testing scenario, final model selection is complicated by the more liberal *n*-locus strategy of PT. While the final goal of MDR is hypothesis generation, and the user may prefer the risk of false positives to the risk of missing a true signal, it is recommended that significance levels be assigned to one or more models from the final set using the omnibus permutation distribution, and not using corresponding *n*-locus tests.

While these results are most immediately applicable to genetic epidemiologists using MDR, they may generalize to any computational method that involves PT. Additionally, as MDR gains acceptance and becomes more widely used, it is important that the consequences of alternative permutation strategies should be explored and understood. Recent work is also implementing alternative hypothesis testing strategies for MDR that are computationally feasible for extremely large-scale datasets [22].

## Abbreviations

MDR: Multifactor Dimensionality Reduction; PT: Permutation testing.

## Competing interests

The authors declare that they have no competing interests.

## Authors' contributions

AAM designed the study, performed the data analysis, and wrote the manuscript.

## Supplementary Material

Additional file 1 **Methods description.** Detail additional file provides detailed descriptions of the methods, data simulation and data analysis performed in the current study. Click here for file
