# Supplementary material for: The effect of alternative permutation testing strategies on the performance of multifactor dimensionality reduction
Source: BMC Res Notes. 2008 Dec 30;1:139. doi: 10.1186/1756-0500-1-139 (PMC2631601; doi:10.1186/1756-0500-1-139)
Supplement: Additional file 1 — Methods description. Detail additional file provides detailed descriptions of the methods, data simulation and data analysis performed in the current study. [file 1756-0500-1-139-S1.doc]

**Methods Description**

**Multifactor Dimensionality Reduction (MDR)**

In step one, the data set is divided into equal partitions for cross-validation [10]. Implementation of both 10-fold and 5-fold cross-validation in MDR has been shown to have equivalent power to detect gene-gene interactions [22]. In the case of five-fold cross-validation, the training set is comprised of 4/5 of the data, while the testing set is comprised of the remaining 1/5 of the data. In this study, all analyses were performed with both 10-fold and 5-fold cross validation. The results were equivalent, so only the results of the 5-fold cross-validation analyses are reported here.

In step two, a set of *n* factors is selected and an exhaustive list of all combinations of factors is then created. Third, these *n* factors are arranged in contingency tables with all possible multifactor combinations as individual cells and the cases and controls are counted for each locus. In step four, the ratio of cases to controls within each contingency table cell is calculated. Each cell is then labeled as “high risk” or “low risk” based on the comparison of that ratio to a threshold of one (since one would expect an equal number of cases and controls under the null hypothesis of no association).

In the next step, the classification error for each model is calculated as the number of individuals misclassified by the high-risk/low-risk model. The model with the lowest classification error is selected as the best *n* locus model and that model is then evaluated against the testing set to calculate prediction error (the proportion of individuals misclassified by the model in the testing set). This entire procedure is repeated for each partition of the data to generate an average classification error and prediction error for the model over the entire dataset. Among all models created, the one model with the maximum cross-validation consistency is chosen. Cross-validation consistency counts the number of times a particular model is identified across the cross-validation sets of data. The higher the cross-validation consistency is, the stronger the evidence for the model. This process is iterated for all 1, 2, … *n* loci combinations that are computationally feasible. A model is chosen for each number of loci considered; so a best one-locus model, two-locus model, three-locus model, etc is selected.

Traditionally, one overall final model is then chosen from this set of models. This final model is selected based upon minimization of prediction error and maximization of cross-validation consistency. When prediction error and cross-validation consistency indicate different models, parsimony is used to choose the simplest model [10]. More recently, emphasis has been placed on significance testing the final model at each level of interaction. In the current study, results are shown using this second approach – testing the best model at each level of interaction (for both types of permutation testing described below).

Once a final model(s) is chosen, permutation testing is used to assess the statistical significance of the hypothesis generated [18]. Randomizing the disease status labels for all individuals in the dataset creates multiple permuted datasets. Typically, one thousand randomized datasets are generated. The entire MDR procedure is repeated for each randomized dataset. The best model is extracted for each random data set (as described above), generating a distribution of one thousand prediction errors that could be expected by chance alone. The significance of the final model is determined by comparing the prediction error of the final model to the random distribution. A p-value is extracted for the final model by its location in this empirical distribution.

**Permutation Testing**

MDR implements a data permutation testing procedure, such that randomizing the disease status labels, while maintaining the multifactor matrix, creates multiple permuted datasets. As mentioned above, typically one thousand randomized datasets are generated and the entire MDR procedure is repeated for each.

In the case of omnibus permutation testing, the best overall model is extracted for each random dataset as described above. This generates a distribution of one thousand prediction errors that could be expected by chance alone. The significance of the final model is determined by comparing the prediction error of the final model to the distribution. A p-value is extracted for the model by its location in this empirical distribution.

In the case of *n*-locus permutation testing, the best model for each *n* level of interaction is extracted to generate each *n*-level distribution. Thus, if single locus through five-way interactions were evaluated, five separate distributions would be created from the one thousand datasets. For each *n*-locus model produced from the original dataset, significance is determined by comparing the prediction error to the corresponding *n*-locus empirical distribution. Again, the empirical p-value is determined by the theoretical location of the real prediction error in the random distribution for the particular level of interaction.

For both types of permutation testing, a prediction error that falls in the top 5% of the appropriate random distribution is considered statistically significant.

**Data Simulations**

Two different minor allele frequency scenarios were chosen for the simulations in the current study: 0.2 and 0.4. A range of heritability values was selected for simulation, including 0.5%, 1%, 1.5%, 2%, and 3%. Roughly, heritability describes the proportion of the total phenotype/disease that is due to genetic effects. More specifically, the exact heritability calculations used can be found in Culverhouse et al [23]. These heritability values represent a very low range of “worst case scenario” to test the lower limits of the method. It is assumed that a method that can find such minimal effects should have greater power to find more substantial effects.

A range of functional interacting loci was simulated using penetrance functions. Penetrance functions define the probability of disease given a particular genotype combination to model the relationship between genetic variations and disease risk. The range of functional loci selected included two, three, four, and five-locus interaction models. All penetrance functions used in the study are available from the author upon request.

Datasets were simulated using software described by Moore et al [24]. All possible combinations of allele frequency, heritability, and interacting loci were modeled, resulting in 40 models. One hundred datasets were generated for each model, resulting in 4000 datasets. Each dataset included 400 total individuals – 200 cases and 200 controls. While the number of functional loci present varied between models, the total number of SNPs was constant in each dataset (10 loci per individual).

In addition to the 40 disease models simulated, a null model was also simulated to test the false positive rate of the method in the complete absence of any genetic signal. The null model was simulated without a penetrance function, so that there was no association between an individual’s genotype and affection status. A total of 10 non-functional SNPs were generated for each individual. One hundred datasets were generated with 200 cases and 200 controls per dataset.

**Data Analysis**

A Linux version of the MDR software was used for data analysis (compiled and benchmarked on a PC with a 600 MHz Pentium-III running Red Hat 2.2.5-15, written in C and compiled with the GNU C compiler). MDR software is currently distributed in a JAVA version with a graphical user interface or in a C library. The most current open-source versions are available at [www.epistasis.org/mdr.html](http://www.epistasis.org/mdr.html). MDR has also been added to Weka-CG which is available from the same website.

Each dataset was analyzed for all single-locus through five-locus combinations. All analyses were performed implementing both 5-fold and 10-fold cross-validation. Identical trends were found for both *k*-fold levels of cross-validation, so only the results for 5-fold cross-validation are shown here.

As mentioned previously, one hundred datasets were created for each epistasis model. Because each of the datasets was generated under the same genetic model, for our power and false positive estimates, one permutation distribution was generated for each model. To ensure that there was no difference in the null distribution created for each simulated dataset generated under one model, one hundred permutation tests were performed for one epistasis model (2-locus interaction with a 0.2 minor allele frequency and 5% heritability) and the null distributions were compared. The results of a Kolgolmorov-Smirnoff test (using STATA v.9) revealed that there is no significant difference between the null distributions of the one hundred simulated datasets (p= 0.9).
